# The AF4-MLL fusion transiently augments multilineage hematopoietic engraftment but is not sufficient to initiate leukemia in cord blood CD34^+^ cells

**DOI:** 10.18632/oncotarget.19567

**Published:** 2017-07-26

**Authors:** Cristina Prieto, Rolf Marschalek, Alessa Kühn, Adelheid Bursen, Clara Bueno, Pablo Menéndez

**Affiliations:** ^1^ Department of Biomedicine, Josep Carreras Leukemia Research Institute, School of Medicine, University of Barcelona, Barcelona, Spain; ^2^ Institute of Pharmaceutical Biology/DCAL, Goethe-University, Frankfurt, Germany; ^3^ Centro de Investigación Biomédica en Red de Cancer (CIBERONC), Barcelona, Spain; ^4^ Instituciò Catalana de Recerca i Estudis Avançats, Barcelona, Spain

**Keywords:** AF4-MLL, CD34 HSPCs, B cell acute lymphoblastic leukemia, leukemogenesis

## Abstract

The translocation t(4;11)(q21;q23) is the hallmark genetic abnormality associated with infant pro-B acute lymphoblastic leukemia (B-ALL) and has the highest frequency of rearrangement in Mixed-lineage leukemia (MLL) leukemias. Unlike other MLL translocations, MLL-AF4-induced proB-ALL is exceptionally difficult to model in mice/humans. Previous work has investigated the relevance of the reciprocal translocation fusion protein AF4-MLL for t(4;11) leukemia, finding that AF4-MLL is capable of inducing proB-ALL without requirement for MLL-AF4 when expressed in murine hematopoietic stem/progenitor cells (HSPCs). Therefore, AF4-MLL might represent a key genetic lesion contributing to t(4;11)-driven leukemogenesis. Here, we aimed to establish a humanized mouse model by using AF4-MLL to analyze its transformation potential in human cord blood-derived CD34^+^ HSPCs. We show that AF4-MLL-expressing human CD34^+^ HSPCs provide enhanced long-term hematopoietic reconstitution in primary immunodeficient recipients but are not endowed with subsequent self-renewal ability upon serial transplantation. Importantly, expression of AF4-MLL in primary neonatal CD34^+^ HSPCs failed to render any phenotypic or hematological sign of disease, and was therefore not sufficient to initiate leukemia within a 36-week follow-up. Species-specific (epi)-genetic intrinsic determinants may underlie the different outcome observed when AF4-MLL is expressed in murine or human HSPCs.

## INTRODUCTION

The translocation t(4;11)(q21;q23) encodes the chimeric proteins mixed-lineage leukemia (MLL)-AF4 and AF4-MLL and is the hallmark genetic abnormality associated with infant pro-B acute lymphoblastic leukemia (B-ALL), which has a dismal prognosis [1, 2]. Our understanding of t(4;11)-mediated transformation is limited, and unlike other MLL fusions, MLL-AF4-induced leukemogenesis has been difficult to model. Current murine and humanized disease models do not faithfully recapitulate the pathogenesis/phenotype [3-5]. It has been claimed that the absence of a suitable t(4;11) disease model is the result of targeting a cell in a wrong developmental stage, or that the impact of secondary hits has not been properly addressed. Very recently, Lin *et al* [6] fused human MLL to murine Af4, resulting in a human-mouse chimeric fusion gene that produced high-titer retrovirus facilitating efficient transduction of human CD34^+^ cells, thereby generating the first “faithful” model of t(4;11) pro-B ALL recapitulating key immunophenotypic/molecular aspects of the disease.

Most chromosomal translocations that have been studied in cancer require only one fusion product for transformation, and in many human cancers the reciprocal fusion is not consistently expressed [7, 8]. The relevance of the reciprocal product AF4-MLL in t(4;11) leukemia has been investigated in previous studies. AF4-MLL fusion protein was capable of inducing B-ALL in mice without requirement of MLL-AF4, indicating that it might represent a key genetic lesion contributing to t(4;11)-driven leukemogenesis [5]. Here, we have investigated in a human stem cell context whether ectopic expression of *AF4-MLL* contributes to transform cord blood (CB)-derived CD34^+^ HSPCs. Our data indicate that AF4-MLL transiently enhances long-term hematopoietic reconstitution in immunodeficient mice, but is not sufficient to initiate leukemia in primary neonatal CD34^+^HSPCs.

## RESULTS AND DISCUSSION

### Enforced expression of *AF4-MLL* in CB-derived CD34^+^HSPCs transiently augments multilineage hematopoietic engraftment and facilitates homing of CD34^+^ HSPCs

**Figure 1:**
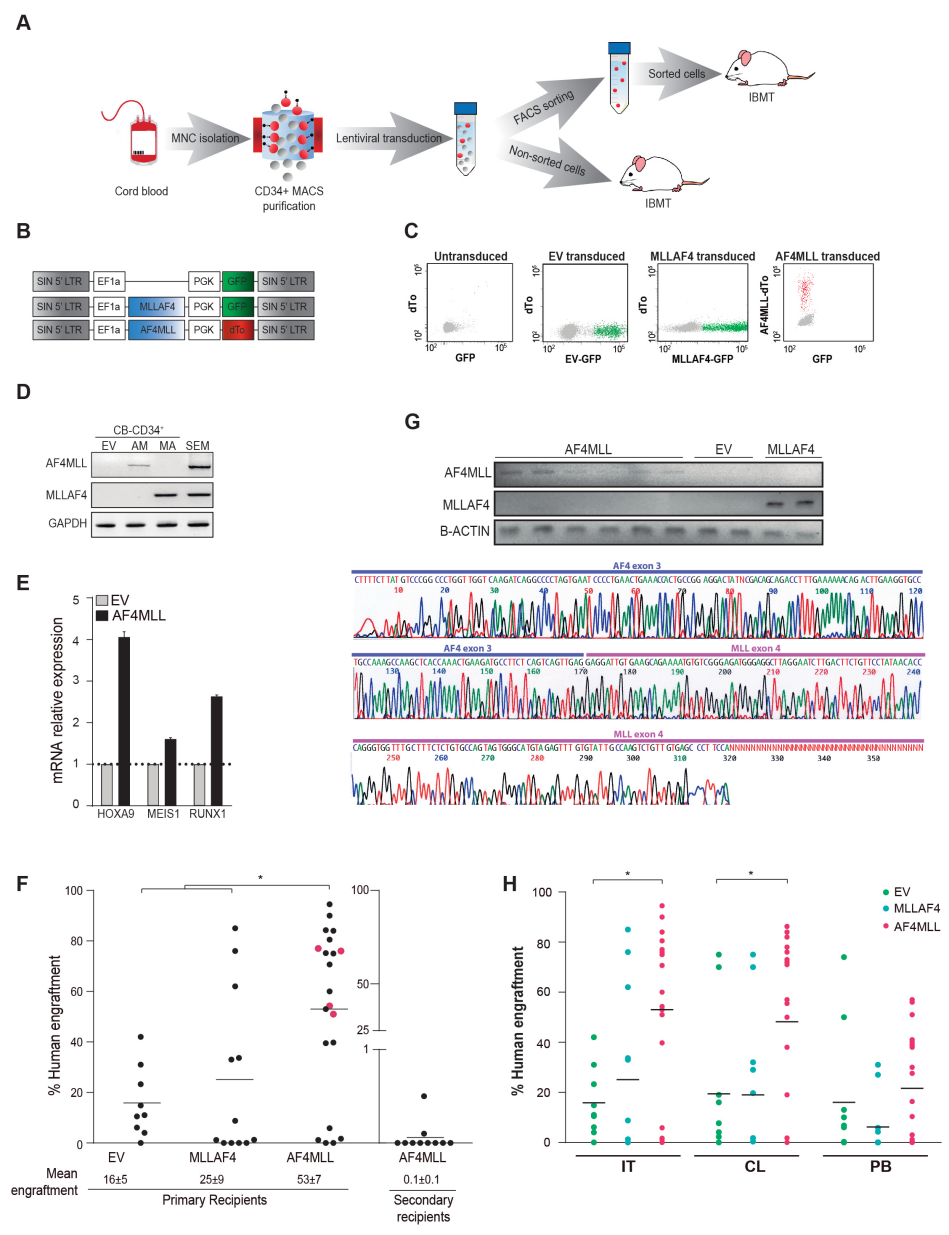
Expression of *AF4-MLL* enhances hematopoietic engraftment of CB-CD34^+^ HSPCs **A.** Outline of the experimental design. **B.** Schematic representation of the lentivectors used. AF4-MLL and MLL-AF4 vectors express dTomato and GFP, respectively, as reporter. **C.** Representative flow cytometry plots showing FACS purification of transduced cells: GFP^+^ cells (green) in *MLL-AF4*-transduced, dTo^+^ cells (red) in *AF4-MLL*-transduced CD34^+^ HSPCs. Mock-transduced cells (left panel) were used as a reference control. **D.** RT-PCR confirming ectopic expression of *AF4-MLL* and *MLL-AF4* in transduced CB-CD34^+^ cells. **E.** RT-qPCR confirming upregulation of the AF4-MLL downstream effectors *HOXA9*, *MEIS1* and *RUNX1* in transduced cells. **F.** Levels of long-term (36 weeks) hematopoietic engraftment of CB-CD34^+^ expressing *AF4-MLL* or *MLL-AF4* (*n* = 42 mice). Note the very limited hematopoietic engraftment of *AF4-MLL*-expressing CB-CD34^+^ upon serial (secondary) xenotransplantation (*n* = 12 mice from 4 independent experiments corresponding to primografts shown in pink). **G.** *Upper panel:* RT-PCR confirming stable ectopic expression of *AF4-MLL* and *MLL-AF4* in xenografts recovered 36 weeks after transplantation. *Lower panel*: Sanger sequencing verifying the AF4-MLL PCR product. **H.** Hematopoietic engraftment in the injected tibia (IT), the contralateral (CL) tibia and the PB of EV, MLL-AF4 and AF4-MLL primografts 36 weeks after transplantation. MNC: Mononuclear cells; EV: Empty vector; IBMT: Intra-bone marrow transplantation.

To assess the developmental impact of AF4-MLL in human HSPCs, CB-derived CD34^+^ cells were MACS-isolated (purity>95%) and transduced with a pRRL-lentivector expressing: (i) GFP reporter (EV), (ii) *MLL-AF4*-GFP and (iii) *AF4-MLL*-dTomato (Figure 1A-1B). Correct reporter (GFP, dTo) and transgene (*MLL-AF4* and *AF4-MLL*) expression in HSPCs was confirmed by flow cytometry (Figure 1C) and RT-PCR (Figure 1D) 3-5 days after transduction. Importantly, ectopic expression of AF4-MLL resulted in 2 to 4-fold upregulation of the master downstream effectors *HOXA9, MEIS1* and *RUNX1* (Figure 1E). To determine whether AF4-MLL regulates HSPCs *in vivo*, purified CD34^+^ HSPCs were transduced with EV, MLL-AF4 or AF4-MLL, sorted based on reporter expression (Figure 1A) and 30, 000 cells were intra-bone marrow transplanted (IBMT) into irradiated (2.5 Gy) 8-10-week-old NSG mice (*n* = 45). In two experiments 300, 000 infected CD34^+^ HSPCs were transplanted without previous purification of transduced cells, rendering a similar outcome. Animals were monitored throughout the experiment and no mouse showed any sign of disease after 36 weeks. Enforced expression of *MLL* fusions, particularly *AF4-MLL*, enhanced hematopoietic engraftment 2- to 3-fold as compared with EV-transduced cells (25% in MLL-AF4 and 53% in AF4-MLL vs 16% in EV; Figure 1F), indicating that AF4-MLL sustains robust engraftment. The ectopic expression of *MLL-AF4* and *AF4-MLL* was confirmed by RT-PCR in bone marrow (BM) cells derived from engrafted animals (Figure 1G). However, AF4-MLL failed to promote further self-renewal upon serial transplantation; the enhanced AF4-MLL-mediated hematopoietic reconstitution observed in primary recipients was transient, and was lost in secondary recipients (*n* = 12 secondary mice from *n* = 4 primary mice; Figure 1F).

IBMT provides the opportunity to assess migration of transplanted CD34^+^ cells *in vivo*. Accordingly, the migration ability of transplanted EV-, MLL-AF4- and AF4-MLL-CD34^+^ cells was assessed by analyzing the level of chimerism in injected tibiae (IT), non-injected tibia (contralateral, CL), spleen and peripheral blood (PB). CD34^+^ HSPCs were capable of migrating to and colonizing other hematopoietic sites in all the animals (Figure 1H). However, the levels of chimerism in CL and PB were 2-fold higher in NSG mice transplanted with *AF4-MLL*-transduced CD34*+* cells. Thus, enforced expression of *AF4-MLL* transiently augments multilineage hematopoietic engraftment and facilitates homing of CD34^+^ HSPCs.

### Expression of *AF4-MLL* in CB-derived CD34^+^ HSPCs fails to initiate leukemogenesis *in vivo*

**Figure 2:**
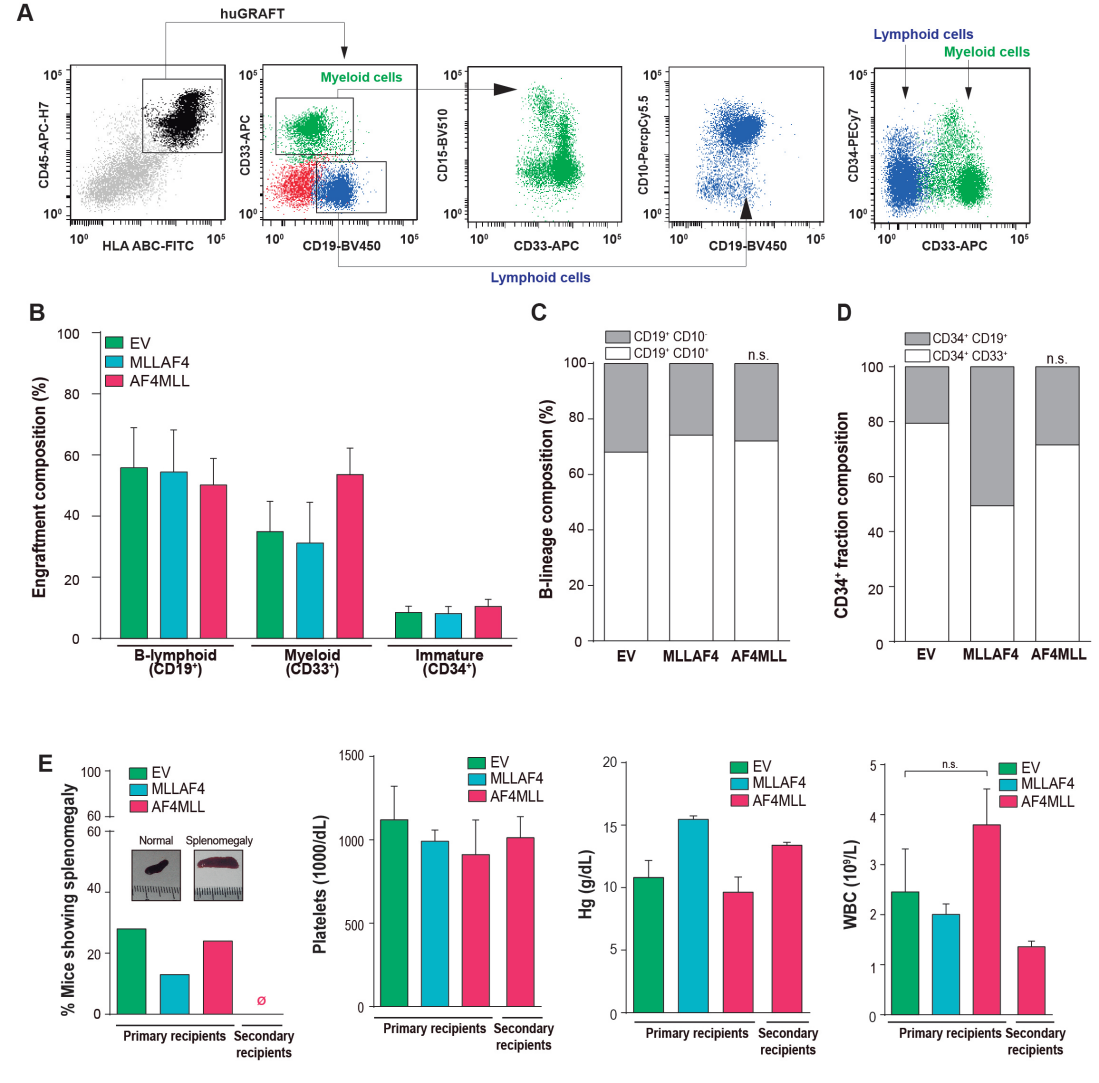
*AF4-MLL* is not sufficient to initiate leukemia in CB-CD34^+^ HSPCs **A.** Representative flow cytometry analysis of chimeric mice. Human engraftment (black) is identified as HLA.ABC^+^ CD45^+^ and includes lymphoid CD19^+^ cells (blue), comprising pre-B (CD10^+^) and pro-B (CD10^-^) fractions, myeloid CD33^+^/CD15^-^ or CD33^+^/CD15^+^ cells (green) and CD34^+^ immature cells (either lymphoid- (CD19^+^) or myeloid-committed (CD33^+^)). **B.** Graft composition confirming normal multilineage engraftment in primografts. **C.** CD19^+^ graft analysis demonstrating normal, non-leukemic pro-B (CD10^-^) to pre-B (CD10^+^) B cell differentiation. **D.** Normal engraftment composition of the immature CD34^+^ fraction including B cell progenitors (CD34^+^CD19^+^; 55-80%) and myeloid progenitors (CD34^+^CD33^+^; 20-45%) (*n* = 42 mice). **E.** *Left panel*, percentage of primografts showing splenomegaly (spleen weight >0.1g) within the indicated genotypes (*n* = 56). Macroscopic images comparing normal *vs* enlarged spleens are shown. *Middle-right panels*, platelet, hemoglobin and WBC counts analyzed in the indicated primary and secondary recipients, revealing no sign of leukemia in reconstituted mice.

We next characterized the composition of the human graft by FACS (Figure 2A, Supplementary Figure 1). Similar multilineage repopulation was always observed in all engrafted mice, irrespective of the expression of *MLL-AF4* or *AF4-MLL* and the tissue analyzed (Figure 2B, Supplementary Figure S1). The graft was consistently lymphoid-biased (CD45^+^CD19^+^;∼60%), followed by CD45^+^CD33^+^ myeloid cells (∼40%) and CD45^+^CD34^+^ immature cells (∼10%) (Figure 2Bl). Because leukemic blasts in t(4;11)^+^ B-ALL are characterized by a CD34^+^CD19^+^CD10^-^ pro-B phenotype, we further analyzed the phenotype of the CD45^+^CD19^+^ B-cell graft and found that ∼25% was composed by pro-B cells and mature B-cells (CD19^+^CD10^-^) coexisting with a predominant pre-B cell population (CD19^+^CD10^+^CD34^-^;∼75%) (Figure 2C). Similarly, the engraftment of CD34+ immature cells was predominantly myeloid (CD33^+^) in mice transplanted with EV- or AF4-MLL-transduced CD34^+^ cells (Figure 2D). A comparable engraftment composition was observed in a cohort (n = 3) of animals sacrificed 12 weeks post-transplantation (Supplementary Figure 2). Thus, normal (non-leukemic) B-cell development and engraftment kinetics were observed in mice transplanted with EV, MLL-AF4 and AF4-MLL CD34^+^ cells.

To further confirm that AF4-MLL did not contribute to leukemia initiation, all the animals lacking disease signals after 36 weeks were sacrificed, and neither splenomegaly (Figure 2E, left panel) nor hepatomegaly was particularly associated with the AF4-MLL genotype. Moreover, platelet counts, hemoglobin levels and leukocyte counts (WBC) were normal/similar between conditions (Figure 2E, middle-right panels). Importantly, secondary recipients of primary mice engrafted with AF4-MLL-expressing CD34^+^ cells had no sign of splenomegaly, anemia or leukocytosis according to the limited engraftment observed upon serial transplantation (Figure 2E). Collectively, the results show that enforced expression of *AF4-MLL* (or *MLL-AF4*) was not sufficient for leukemogenesis.

We acknowledge some limitations in our study. First, the *AF4-MLL* fusion is 8 kb in length, so the expressing lentivector may produce low viral titers. Our *AF4-MLL*-expressing lentivirus titer was consistently ∼106 infective particles, which suffices to infect target cells at a reasonable MOI, rendering stable transcript expression (sequenced) *in vitro* (Figure 1C, 1D) and *in vivo* (Figure 1G) even 36 weeks after transplantation. The same transcript was successfully expressed in a “safe harbor” [12] using CRISPR/Cas9 genome-editing, suggesting that the inability of AF4-MLL to initiate leukemia may not be attributed to the absence of transcript expression. Second, it is plausible that co-expression of *MLL-AF4* and *AF4-MLL* is required in the same target cell for leukemia initiation. Lentiviral expression of both cassettes in the same CD34^+^ HSPC is challenging, but a CRISPR/Cas9-mediated recreation of the t(4;11) by producing both allele-specific MLL fusions would undoubtedly open new avenues in future research [13]. Alternatively, the generation of an *AF4-MLL* knock-in mouse, where *AF4-MLL* is conditionally expressed in the context of the *AF4* locus, could be a valuable model to test whether *AF4-MLL* is needed for leukemia onset. Arguments in favor for the need for *AF4-MLL* are: (i) the extreme rapid onset of disease after birth; the existing mouse data with *AF4-MLL* alone [5] and its proposed function [14]. Arguments against the need for *AF4-MLL* to be co-expressed with *MLL-AF4* for B-ALL initiation are: (i) at least one third of t(4;11)^+^ patients display complex genetic rearrangements and do not express particularly *AF4-MLL* [15, 16]; (ii) ablation of AF4-MLL in leukemic cell lines seems dispensable for cell growth in short term siRNA experiments [14]. Therefore, AF4-MLL may well cooperate with MLL-AF4 in leukemia phenotype, aggressiveness or therapy-resistance rather than in leukemia onset/initiation.

## MATERIALS AND METHODS

The study was IRB-approved by the Clinic Hospital of Barcelona and CB units from healthy newborns were accessed from the Barcelona Cord Blood Bank upon signed informed consent.

MACS enrichment of CD34^+^ HSPCs, lentiviral-mediated transduction of MLL-AF4 and AF4-MLL, reporter-based FACS-isolation of transduced cells, serial intra-bone marrow xenotransplantation (IBMT), mouse follow-up, flow cytometric analysis of multi-lineage/multi-organ chimerism, RT-PCR (conditions and primers), and assessment of hematological parameters were done as previously described by our group and others [5, 9-12, 15, 16]. The AF4-MLL fusion cloned in the pRRL lentivector (pRRL-EF1a-AF4-MLL-PGK-dTo) was sequenced as shown in Supplementary Figure 3, which highlights the breakpoint and the start/stop codons.

### Ethics approval and consent to participate

All animal procedures were approved by the local ethical committee (Comité Ético de Experimentación Animal del PRBB (CEEA-PRBB); procedure numbers MDS-08-1060P1 and JMC-07-1001P1-MDS), and met the guidelines of the local (law 32/2007) and European regulations (EU directive n° 86/609, EU decree 2001-486) and the Standards for Use of Laboratory Animals n° A5388-01 (NIH).

## SUPPLEMENTARY MATERIALS FIGURES



## Abbreviations

HSPCs: Hematopoietic Stem and Progenitor Cells
MLL: Mixed lineage leukemia
B-ALL: Pro-B Acute Lymphoblastic Leukemia
MACS: magnetic activated cell sorting
FACS: fluorescence activated cell sorting
IBMT: intra-bone marrow transplantation
CB: cord blood
EV: empty vector
IT: injected tibiae
CL: contralateral
PB: peripheral blood
