# Supplementary material for: The AF4-MLL fusion transiently augments multilineage hematopoietic engraftment but is not sufficient to initiate leukemia in cord blood CD34+ cells
Source: Oncotarget. 2017 Jul 26;8(47):81936–41. doi: 10.18632/oncotarget.19567 (PMC5669860; doi:10.18632/oncotarget.19567)
Supplement: Supplementary file 1 [file oncotarget-08-81936-s001.pdf]

The AF4-MLL fusion transiently augments multilineage hematopoietic engraftment but is not sufficient to initiate leukemia in cord blood CD34+ cells

SUPPLEMENTARY MATERIALS

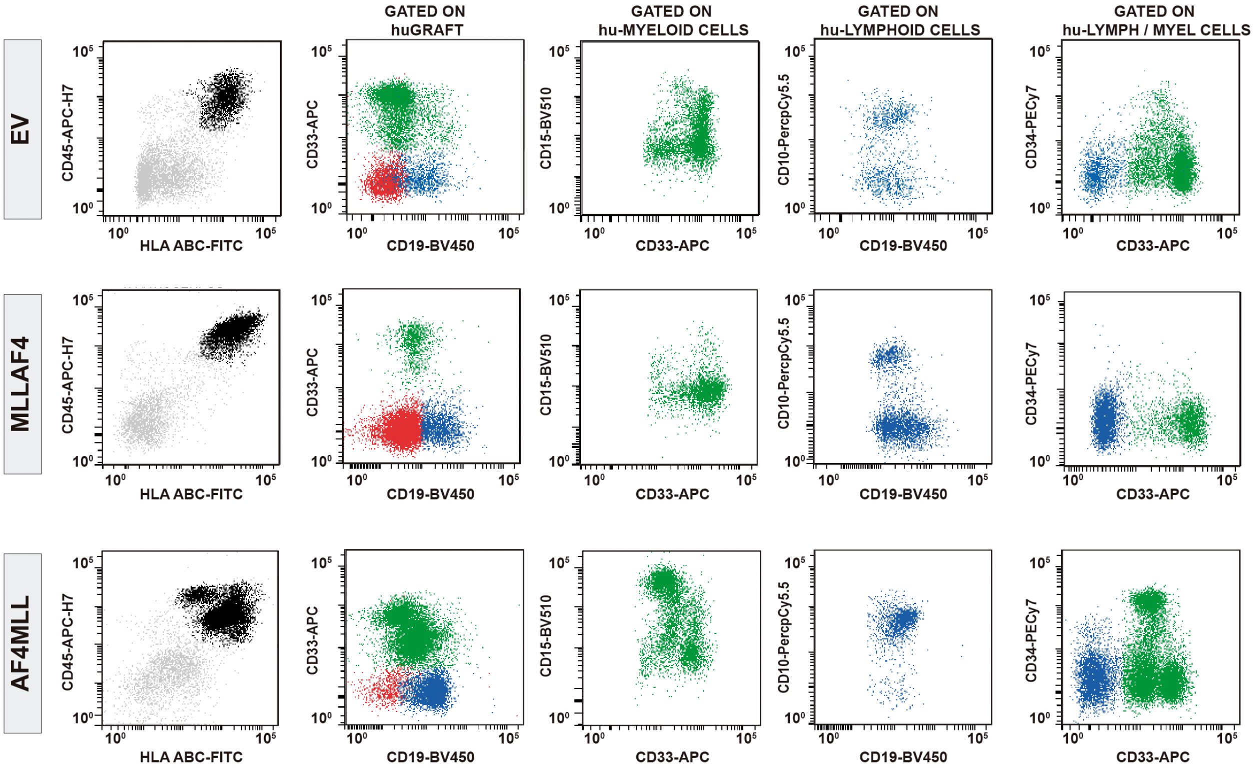

Supplementary Figure 1: Representative flow cytometry analysis of mice transplanted with EV-, MLL-AF4- or AF4-MLL-expressing CD34$^+$ HSPCs. Human engraftment (black) is identified as HLA.ABC$^+$ CD45$^+$ and includes lymphoid CD19$^+$ cells (blue), comprising pre-B (CD10$^+$) and pro-B (CD10$^-$) fractions, myeloid CD33$^+$/CD15$^-$ and CD33$^+$/CD15$^+$ cells (green) and a few CD34$^+$ immature cells (either lymphoid- (CD19$^+$) or myeloid-committed (CD33+)).

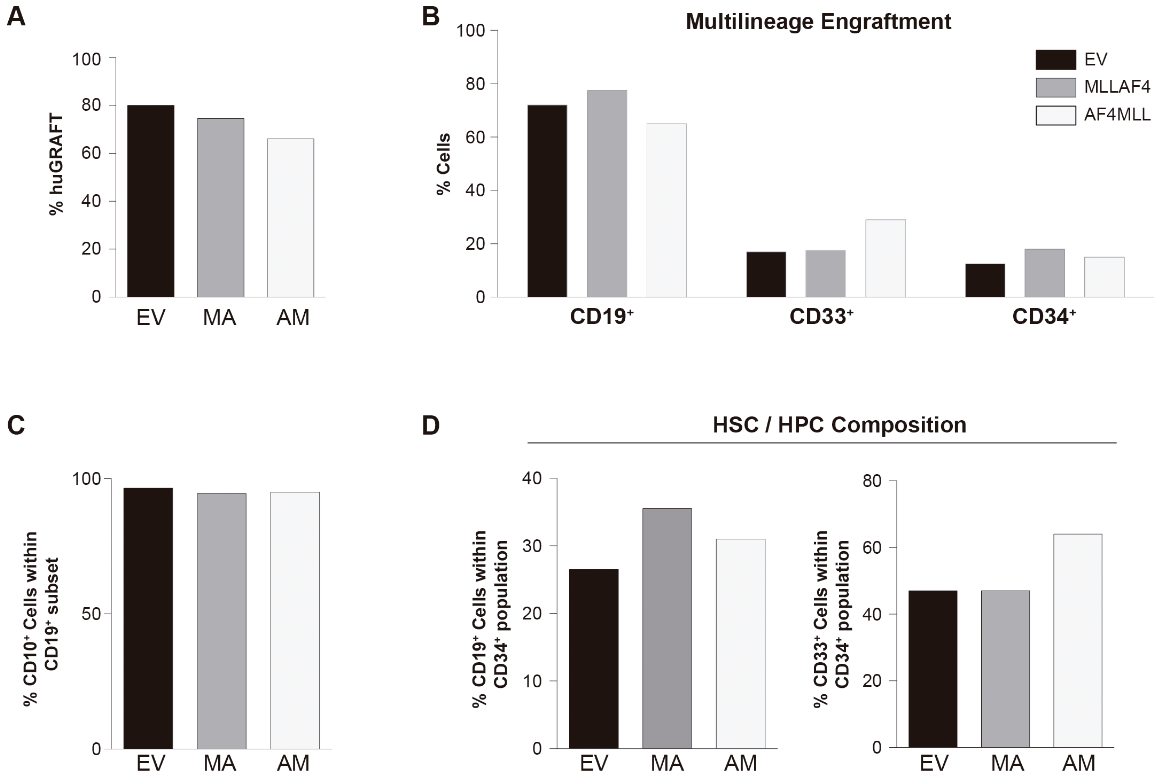

Supplementary Figure 2: Twelve-week engraftment composition of mice transplanted with EV-, MLL-AF4- or AF4-MLL-expressing CD34^{+} HSPCs. (A) Total engraftment identified as HLA.ABC^{+} CD45^{+}. (B) Multilineage engraftment including CD19^{+} B-lymphoid, CD33^{+} myeloid and CD34^{+}+ immature engraftment. (C) Proportion of CD10^{+} pre-B cells within the CD19^{+} B-lymphoid engraftment. (D) Proportion of CD19^{+} (Band CD33^{+} cells within the CD34^{+} immature engraftment).

AF4-MLL (8739 bp)

Red: AF4 exon 1+AF4 exon 2

Blue: MLL exon 10 to 36

ATG :Start Codon

TAA :Stop Codon

ATGGCAGCCCAGTCAAGTTTGTACAATGACGACAGAAACCTGCTTCGAATTAG
AGAGAAGGAAAGACGCAACCAGGAAGCCCACCAAGAGAAAGAGGCATTTCCT
GAAAAGATTCCCCTTTTTGGAGAGCCCTACAAGACAGCAAAAGGTGATGAGCT
GTCTAGTCGAATACAGAACATGTTGGGAAACTACGAAGAAGTGAAGGAGTTC
CTTAGTACTAAGTCTCACACTCATCGCCTGGATGCTTCTGAAAATAGGTTGGG
AAAGCCGAAATATCCTTTAATTCCTGACAAAGGGAGCAGCATTCCATCCAGCT
CCTTCCACACTAGTGTCCACCACCAGTCCATTCACACTCCTGCGTCTGGACCA
CTTTCTGTTGGCAACATTAGCCACAATCCAAAGATGGCGCAGCCAAGAACTGA
ACCAATGCCAAGTCTCCATGCCAAAAGCTGCGGCCCACCGGACAGCCAGCAC
CTGACCCAGGATCGCCTTGGTCAGGAGGGGTTCGGCTCTAGTCATCACAAGA
AAGGTGACCGAAGAGCTGACGGAGACCACTGTGCTTCGGTGACAGATTCGG
CTCCAGAGAGGGAGCTTTCTCCCTTAATCTCTTTGCCTTCCCCAGTTCCCCCT
TTGTCACCTATACATTCCAACCAGCAAACTCTTCCCCGGACGCAAGGAAGCAG
CAAGGTTCATGGCAGCAGCAATAACAGTAAAGGCTATTGCCCAGCCAAATCTC
CCAAGGACCTAGCAGTGAAAGTCCATGATAAAGAGACCCCTCAAGACAGTTT
GGTGGCCCCTGCCCAGCCGCCTTCTCAGACATTTCCACCTCCCTCCCTCCCC
TCAAAAAGTGTTGCAATGCAGCAGAAGCCCACGGCTTATGTCCGGCCCATGG
ATGGTCAAGATCAGGCCCCTAGTGAATCCCCTGAACTGAAACCACTGCCGGA
GGACTATCGACAGCAGACCTTTGAAAAAACAGACTTGAAAGTGCCTGCCAAA
GCCAAGCTCACCAAACTGAAGATGCCTTCTCAGTCAGTTGAGGAGGATTGTG
AAGCAGAAAATGTGTGGGAGATGGGAGGCTTAGGAATCTTGACTTCTGTTCCT
ATAACACCCAGGGTGGTTTGCTTTCTCTGTGCCAGTAGTGGGCATGTAGAGTT
TGTGTATTGCCAAGTCTGTTGTGAGCCCTTCCACAAGTTTTGTTTAGAGGAGA
ACGAGCGCCCTCTGGAGGACCAGCTGGAAAATTGGTGTTGTCGTCGTTGCAA
ATTCTGTCACGTTTGTGGAAGGCAACATCAGGCTACAAAGCAGCTGCTGGAG
TGTAATAAGTGCCGAAACAGCTATCACCCTGAGTGCCTGGGACCAAACTACC
CCACCAAACCCACAAAGAAGAAGAAAGTCTGGATCTGTACCAAGTGTGTTCG
CTGTAAGAGCTGTGGATCCACAACTCCAGGCAAAGGGTGGGATGCACAGTGG
TCTCATGATTTCTCACTGTGTCATGATTGCGCCAAGCTCTTTGCTAAAGGAAA
CTTCTGCCCTCTCTGTGACAAATGTTATGATGATGATGACTATGAGAGTAAGA
TGATGCAATGTGGAAAGTGTGATCGCTGGGTCCATTCCAAATGTGAGAATCTT
TCAGGTACAGAAGATGAGATGTATGAGATTCTATCTAATCTGCCAGAAAGTGT
GGCCTACACTTGTGTGAACTGTACTGAGCGGCACCCTGCAGAGTGGCGACTG
GCCCTTGAAAAAGAGCTGCAGATTTCTCTGAAGCAAGTTCTGACAGCTTTGTT
GAATTCTCGGACTACCAGCCATTTGCTACGCTACCGGCAGGCTGCCAAGCCT

CCAGACTTAAATCCCGAGACAGAGGAGAGTATACCTTCCCGCAGCTCCCCCG
AAGGACCTGATCCACCAGTTCTTACTGAGGTCAGCAAACAGGATGATCAGCA
GCCTTTAGATCTAGAAGGAGTCAAGAGGAAGATGGACCAAGGGAATTACACA
TCTGTGTTGGAGTTCAGTGATGATATTGTGAAGATCATTCAAGCAGCCATTAAT
TCAGATGGAGGACAGCCAGAAATTAAAAAAGCCAACAGCATGGTCAAGTCCTT
CTTCATTCGGCAAATGGAACGTGTTTTTCCATGGTTCAGTGTCAAAAAGTCCA
GGTTTTGGGAGCCAAATAAAGTATCAAGCAACAGTGGGATGTTACCAAACGCA
GTGCTTCCACCTTCACTTGACCATAATTATGCTCAGTGGCAGGAGCGAGAGG
AAAACAGCCACACTGAGCAGCCTCCTTTAATGAAGAAAATCATTCCAGCTCCC
AAACCCAAAGGTCCTGGAGAACCAGACTCACCAACTCCTCTGCATCCTCCTAC
ACCACCAATTTTGAGTACTGATAGGAGTCGAGAAGACAGTCCAGAGCTGAAC
CCACCCCCAGGCATAGAAGACAATAGACAGTGTGCGTTATGTTTGACTTATGG
TGATGACAGTGCTAATGATGCTGGTCGTTTACTATATATTGGCCAAAATGAGT
GGACACATGTAAATTGTGCTTTGTGGTCAGCGGAAGTGTTTGAAGATGATGAC
GGATCACTAAAGAATGTGCATATGGCTGTGATCAGGGGCAAGCAGCTGAGAT
GTGAATTCTGCCAAAAGCCAGGAGCCACCGTGGGTTGCTGTCTCACATCCTG
CACCAGCAACTATCACTTCATGTGTTCCCGAGCCAAGAACTGTGTCTTTCTGG
ATGATAAAAAAGTATATTGCCAACGACATCGGGATTTGATCAAAGGCGAAGTG
GTTCCTGAGAATGGATTTGAAGTTTTCAGAAGAGTGTTTGTGGACTTTGAAGG
AATCAGCTTGAGAAGGAAGTTTCTCAATGGCTTGGAACCAGAAAATATCCACA
TGATGATTGGGTCTATGACAATCGACTGCTTAGGAATTCTAAATGATCTCTCC
GACTGTGAAGATAAGCTCTTTCCTATTGGATATCAGTGTTCCAGGGTATACTG
GAGCACCACAGATGCTCGCAAGCGCTGTGTATATACATGCAAGATAGTGGAG
TGCCGTCCTCCAGTCGTAGAGCCGGATATCAACAGCACTGTTGAACATGATG
AAAACAGGACCATTGCCCATAGTCCAACATCTTTTACAGAAAGTTCATCAAAA
GAGAGTCAAAACACAGCTGAAATTATAAGTCCTCCATCACCAGACCGACCTCC
TCATTCACAAACCTCTGGCTCCTGTTATTATCATGTCATCTCAAAGGTCCCCAG
GATTCGAACACCCAGTTATTCTCCAACACAGAGATCCCCTGGCTGTCGACCGT
TGCCTTCTGCAGGAAGTCCTACCCCAACCACTCATGAAATAGTCACAGTAGGT
GATCCTTTACTCTCCTCTGGACTTCGAAGCATTGGCTCCAGGCGTCACAGTAC
CTCTTCCTTATCACCCCAGCGGTCCAAACTCCGGATAATGTCTCCAATGAGAA
CTGGGAATACTTACTCTAGGAATAATGTTTCCTCAGTCTCCACCACCGGGACC
GCTACTGATCTTGAATCAAGTGCCAAAGTAGTTGATCATGTCTTAGGGCCACT
GAATTCAAGTACTAGTTTAGGGCAAAACACTTCCACCTCTTCAAATTTGCAAAG
GACAGTGGTTACTGTAGGCAATAAAAACAGTCACTTGGATGGATCTTCATCTT
CAGAAATGAAGCAGTCCAGTGCTTCAGACTTGGTGTCCAAGAGCTCCTCTTTA
AAGGGAGAGAAGACCAAAGTGCTGAGTTCCAAGAGCTCAGAGGGATCTGCAC
ATAATGTGGCTTACCCTGGAATTCCTAAACTGGCCCCACAGGTTCATAACACA
ACATCTAGAGAACTGAATGTTAGTAAAATCGGCTCCTTTGCTGAACCCTCTTC
AGTGTCGTTTTCTTCTAAAGAGGCCCTCTCCTTCCCACACCTCCATTTGAGAG
GGCAAAGGAATGATCGAGACCAACACACAGATTCTACCCAATCAGCAAACTC
CTCTCCAGATGAAGATACTGAAGTCAAAACCTTGAAGCTATCTGGAATGAGCA
ACAGATCATCCATTATCAACGAACATATGGGATCTAGTTCCAGAGATAGGAGA

CAGAAAGGGAAAAAATCCTGTAAAGAAACTTTCAAAGAAAAGCATTCCAGTAA
ATCTTTTTTGGAACCTGGTCAGGTGACAACTGGTGAGGAAGGAAACTTGAAGC
CAGAGTTTATGGATGAGGTTTTGACTCCTGAGTATATGGGCCAACGACCATGT
AACAATGTTTCTTCTGATAAGATTGGTGATAAAGGCCTTTCTATGCCAGGAGT
CCCCAAAGCTCCACCCATGCAAGTAGAAGGATCTGCCAAGGAATTACAGGCA
CCACGGAAACGCACAGTCAAAGTGACACTGACACCTCTAAAAATGGAAAATGA
GAGTCAATCCAAAAATGCCCTGAAAGAAAGTAGTCCTGCTTCCCCTTTGCAAA
TAGAGTCAACATCTCCCACAGAACCAATTTCAGCCTCTGAAAATCCAGGAGAT
GGTCCAGTGGCCCAACCAAGCCCCAATAATACCTCATGCCAGGATTCTCAAA
GTAACAACTATCAGAATCTTCCAGTACAGGACAGAAACCTAATGCTTCCAGAT
GGCCCCAAACCTCAGGAGGATGGCTCTTTTAAAAGGAGGTATCCCCGTCGCA
GTGCCCGTGCACGTTCTAACATGTTTTTTGGGCTTACCCCACTCTATGGAGTA
AGATCCTATGGTGAAGAAGACATTCCATTCTACAGCAGCTCAACTGGGAAGAA
GCGAGGCAAGAGATCAGCTGAAGGACAGGTGGATGGGGCCGATGACTTAAG
CACTTCAGATGAAGACGACTTATACTATTACAACTTCACTAGAACAGTGATTTC
TTCAGGTGGAGAGGAACGACTGGCATCCCATAATTTATTTCGGGAGGAGGAA
CAGTGTGATCTTCCAAAAATCTCACAGTTGGATGGTGTTGATGATGGGACAGA
GAGTGATACTAGTGTCACAGCCACAACAAGGAAAAGCAGCCAGATTCCAAAA
AGAAATGGTAAAGAAAATGGAACAGAGAACTTAAAGATTGATAGACCTGAAGA
TGCTGGGGAGAAAGAACATGTCACTAAGAGTTCTGTTGGCCACAAAAATGAG
CCAAAGATGGATAACTGCCATTCTGTAAGCAGAGTTAAAACACAGGGACAAGA
TTCCTTGGAAGCTCAGCTCAGCTCATTGGAGTCAAGCCGCAGAGTCCACACA
AGTACCCCCTCCGACAAAAATTTACTGGACACCTATAATACTGAGCTCCTGAA
ATCAGATTCAGACAATAACAACAGTGATGACTGTGGGAATATCCTGCCTTCAG
ACATTATGGACTTTGTACTAAAGAATACTCCATCCATGCAGGCTTTGGGTGAG
AGCCCAGAGTCATCTTCATCAGAACTCCTGAATCTTGGTGAAGGATTGGGTCT
TGACAGTAATCGTGAAAAAGACATGGGTCTTTTTGAAGTATTTTCTCAGCAGCT
GCCTACAACAGAACCTGTGGATAGTAGTGTCTCTTCCTCTATCTCAGCAGAGG
AACAGTTTGAGTTGCCTCTAGAGCTACCATCTGATCTGTCTGTCTTGACCACC
CGGAGTCCCACTGTCCCCAGCCAGAATCCCAGTAGACTAGCTGTTATCTCAG
ACTCAGGGGAGAAGAGAGTAACCATCACAGAAAAATCTGTAGCCTCCTCTGA
AAGTGACCCAGCACTGCTGAGCCCAGGAGTAGATCCAACTCCTGAAGGCCAC
ATGACTCCTGATCATTTTATCCAAGGACACATGGATGCAGACCACATCTCTAG
CCCTCCTTGTGGTTCAGTAGAGCAAGGTCATGGCAACAATCAGGATTTAACTA
GGAACAGTAGCACCCCTGGCCTTCAGGTACCTGTTTCCCCAACTGTTCCCAT
CCAGAACCAGAAGTATGTGCCCAATTCTACTGATAGTCCTGGCCCGTCTCAGA
TTTCCAATGCAGCTGTCCAGACCACTCCACCCCACCTGAAGCCAGCCACTGA
GAAACTCATAGTTGTTAACCAGAACATGCAGCCACTTTATGTTCTCCAAACTCT
TCCAAATGGAGTGACCCAAAAAATCCAATTGACCTCTTCTGTTAGTTCTACACC
CAGTGTGATGGAGACAAATACTTCAGTATTGGGACCCATGGGAGGTGGTCTC
ACCCTTACCACAGGACTAAATCCAAGCTTGCCAACTTCTCAATCTTTGTTCCCT
TCTGCTAGCAAAGGATTGCTACCCATGTCTCATCACCAGCACTTACATTCCTT
CCCTGCAGCTACTCAAAGTAGTTTCCCACCAAACATCAGCAATCCTCCTTCAG

GCCTGCTTATTGGGGTTCAGCCTCCTCCGGATCCCCAACTTTTGGTTTCAGAA
TCCAGCCAGAGGACAGACCTCAGTACCACAGTAGCCACTCCATCCTCTGGAC
TCAAGAAAAGACCCATATCTCGTCTACAGACCCGAAAGAATAAAAAACTTGCT
CCCTCTAGTACCCCTTCAAACATTGCCCCTTCTGATGTGGTTTCTAATATGACA
TTGATTAACTTCACACCCTCCCAGCTTCCTAATCATCCAAGTCTGTTAGATTTG
GGGTCACTTAATACTTCATCTCACCGAACTGTCCCCAACATCATAAAAAGATCT
AAATCTAGCATCATGTATTTTGAACCGGCACCCCTGTTACCACAGAGTGTGGG
AGGAACTGCTGCCACAGCGGCAGGCACATCAACAATAAGCCAGGATACTAGC
CACCTCACATCAGGGTCTGTGTCTGGCTTGGCATCCAGTTCCTCTGTCTTGAA
TGTTGTATCCATGCAAACTACCACAACCCCTACAAGTAGTGCGTCAGTTCCAG
GACACGTCACCTTAACCAACCCAAGGTTGCTTGGTACCCCAGATATTGGCTCA
ATAAGCAATCTTTTAATCAAAGCTAGCCAGCAGAGCCTGGGGATTCAGGACCA
GCCTGTGGCTTTACCGCCAAGTTCAGGAATGTTTCCACAACTGGGGACATCA
CAGACCCCCTCTACTGCTGCAATAACAGCGGCATCTAGCATCTGTGTGCTCC
CCTCCACTCAGACTACGGGCATAACAGCCGCTTCACCTTCTGGGGAAGCAGA
CGAACACTATCAGCTTCAGCATGTGAACCAGCTCCTTGCCAGCAAAACTGGG
ATTCATTCTTCCCAGCGTGATCTTGATTCTGCTTCAGGGCCCCAGGTATCCAA
CTTTACCCAGACGGTAGACGCTCCTAATAGCATGGGACTGGAGCAGAACAAG
GCTTTATCCTCAGCTGTGCAAGCCAGCCCCACCTCTCCTGGGGGTTCTCCAT
CCTCTCCATCTTCTGGACAGCGGTCAGCAAGCCCTTCAGTGCCGGGTCCCAC
TAAACCCAAACCAAAAACCAAACGGTTTCAGCTGCCTCTAGACAAAGGGAATG
GCAAGAAGCACAATGTTTCCCATTTGCGGACCAGTTCTTCTGAAGCACACATT
CCAGACCAAGAAACGACATCCCTGACCTCAGGCACAGGGACTCCAGGAGCA
GAGGCTGAGCAGCAGGATACAGCTAGCGTGGAGCAGTCCTCCCAGAAGGAG
TGTGGGCAACCTGCAGGGCAAGTCGCTGTTCTTCCGGAAGTTCAGGTGACCC
AAAATCCAGCAAATGAACAAGAAAGTGCAGAACCTAAAACAGTGGAAGAAGA
GGAAAGTAATTTCAGCTCCCCACTGATGCTTTGGCTTCAGCAAGAACAAAAGC
GGAAGGAAAGCATTACTGAGAAAAAACCCAAGAAAGGACTTGTTTTTGAAATT
TCCAGTGATGATGGCTTTCAGATCTGTGCAGAAAGTATTGAAGATGCCTGGAA
GTCATTGACAGATAAAGTCCAGGAAGCTCGATCAAATGCCCGCCTAAAGCAG
CTCTCATTTGCAGGTGTTAACGGTTTGAGGATGCTGGGGATTCTCCATGATGC
AGTTGTGTTCCTCATTGAGCAGCTGTCTGGTGCCAAGCACTGTCGAAATTACA
AATTCCGTTTCCACAAGCCAGAGGAGGCCAATGAACCCCCCTTGAACCCTCA
CGGCTCAGCCAGGGCTGAAGTCCACCTCAGGAAGTCAGCATTTGACATGTTT
AACTTCCTGGCTTCTAAACATCGTCAGCCTCCTGAATACAACCCCAATGATGA
AGAAGAGGAGGAGGTACAGCTGAAGTCAGCTCGGAGGGCAACTAGCATGGA
TCTGCCAATGCCCATGCGCTTCCGGCACTTAAAAAAGACTTCTAAGGAGGCA
GTTGGTGTCTACAGGTCTCCCATCCATGGCCGGGGTCTTTTCTGTAAGAGAAA
CATTGATGCAGGTGAGATGGTGATTGAGTATGCCGGCAACGTCATCCGCTCC
ATCCAGACTGACAAGCGGGAAAAGTATTACGACAGCAAGGGCATTGGTTGCT
ATATGTTCCGAATTGATGACTCAGAGGTAGTGGATGCCACCATGCATGGAAAT
GCTGCACGCTTCATCAATCACTCGTGTGAGCCTAACTGCTATTCTCGGGTCAT
CAATATTGATGGGCAGAAGCACATTGTCATCTTTGCCATGCGTAAGATCTACC

GAGGAGAGGAACTCACTTACGACTATAAGTTCCCCATTGAGGATGCCAGCAA
CAAGCTGCCCTGCAACTGTGGCGCTAAGAAATGCCGGAAGTTCCTAAACTAA

Supplementary Figure 3: primers sequences.
